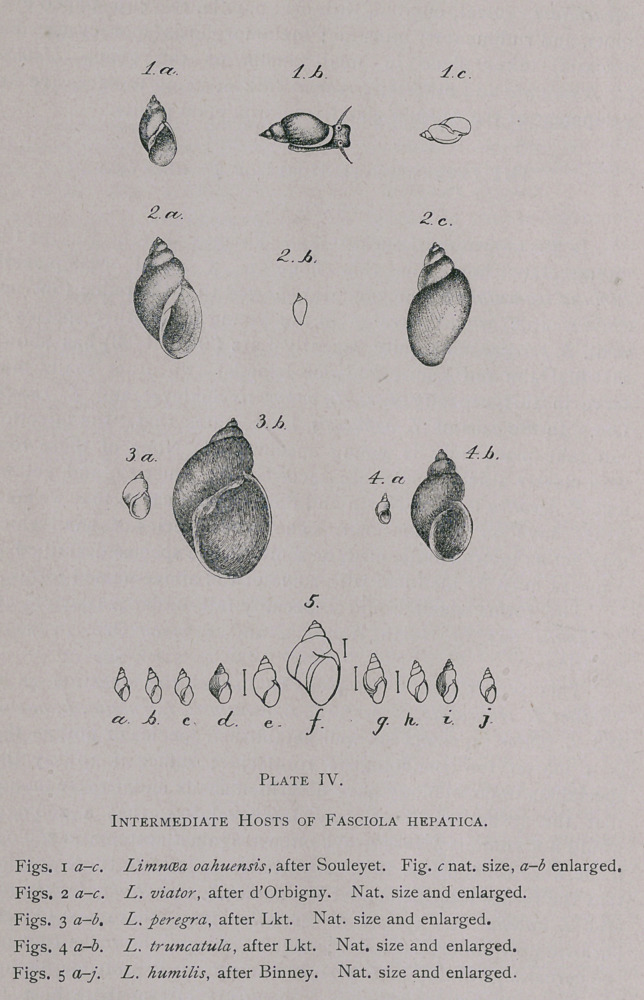# The Anatomy of the Large American Fluke (*Fasciola Magna*)

**Published:** 1894-10

**Authors:** Charles Wardell Stiles

**Affiliations:** Zoȯlogist, Bureau of Animal Industry


THE JOURNAL

OF

COMPARATIVE MEDICINE AND

VETERINARY ARCHIVES.

VOL. XV. OCTOBER, 1894. No. 5.

### THE ANATOMY OF THE LARGE AMERICAN FLUKE (*FASCIOLA MAGNA*), AND A COMPARISON WITH OTHER SPECIES OF THE GENUS FASCIOLA, S.ST.

BY CHAS. WARDELL STILES, PH.D.,

*Zoölogist, Bureau of Animal Industry.*

### CONTAINING ALSO A LIST OF THE CHIEF EPIZOÖTICS OF FASCIOLIASIS (DISTOMATOSIS) AND A BIBLIOGRAPHY OF *FASCIOLA HEPATICA.*

BY ALBERT HASSALL, M.R.C.V.S.

(Continued from page 243.)

### II. **Fasciola hepatica** L., 1746 et 1758.

PLATE III.

This species has been so carefully studied by so many authors that it is useless to repeat the details of the anatomy in this paper. It will suffice for present purposes if the synonymy, hosts, geographical distribution, literature, specific diagnosis and descriptions of the intermediate hosts are given.

PLATE III.

FASCIOLA HEPATICA L.

Fig. 1. American specimen of *F. hepatica* from cattle. Stained preparation to show the internal anatomy. The intestinal system can be seen only in the anterior conical portion. The genital organs can be made out by comparing this figure with Plate I, Fig. 3. In the latter figure, however, the ovary is dextral, in this figure it is sinistral.

SYNONYMY:

1746 et 1758, *Fasciola hepatica* L.;
1782, *Planaria latiuscula* Goeze;
1786, *Distoma hepaticum* Abildg.;
1789, *F. humana* Gmelin;
1803, *F. lanceolata* Rud.;
1845, *Distoma* (*Cladocœlium*) *hepaticum* Duj.;
1845, *Fasciolaria hepatica* (Anonymous);
1884, *Distomata hominis* Taylor;
1889, *Distomum* (*Fasciola*) *hepaticum* R. Lkt.;
1890, *D. caviæ* Sons ;
1892, *Cladocœlium hepaticum* Stossich.

COMMON NAMES:

English —The common liver-fluke, liver-fluke;
German—Leberegel, Leberwurm, Schafegel;
Dutch – Botten, Leverworm;
Danish—Faareflynder;
Swedish —Levermask;
French—Douve hepatique, fasciole;
Italian—Biscuola, distoma epatico;
Spanish—Caracolillo.

HOSTS:

Man (*Homo sapiens*);
Common European squirrel (*Sciurus vulgaris*);
European beaver (*Castor fiber*);
Tame rabbit (*Lepus cuniculus domesticus*);
Wild rabbit (*L. cuniculus ferus*);
Hare (*L. timidus*);
Cat (*Felis domestica*);
Swine (*Sus scrofa*);
Egyptian buffalo (*Bos bubalus*);
Cattle (*B. taurus*);
Argale sheep (*Ovis argali*);
Domestic sheep (*O. aries*);
Goat (*Capra hircus*);
Gazelle (*Gazella dorcas*);
Roe deer (*Capreolus caprea*);
Fallow deer (*Cervus dama*);
Stag (*Cervus elaphus*);
Blue bull (*Bosephalus tragocamelus*);
Virginian deer (*Cariacus virginianus*);

Bactrian camel (*Camelus bactrianus*);
Ass (*Equus asinus*);
Horse (*E. caballus*);
Sword-fish (*Orca gladiator*);*
Great gray kangaroo (*Macropus giganteus*).

*Leuckart once gave me two specimens of a fluke, which I still have in my possession, labelled "Leber, Schwert-fisch." I am unable to distinguish this fluke from *F. hepatica*. I assume that this "Schwert fisch" is *Orca gladiator* rather than *Xiphias gladius*, as all the other hosts of *F. hepatica* are mammals.

Geographical Distribution.—This species is almost cosmopolitan, being recorded from all the countries of Europe; Asia (India); Africa; Australia; Sandwich Islands; South America (Buenos Ayres and elsewhere). In North America we have positive proof of its occurrence in Long Island, N. Y. (Law); Chicago, Ill., cattle came from Texas (Stiles); California (Curtice); Arkansas (Dinwiddie); Texas (Francis, Detmers); Louisiana (Wheeler). "Rot" is recorded in many other localities, but the term "Rot" in American literature covers a multitude of diseases.

### Specific Diagnosis.

*F. hepatica* L. 1746 et 1758.—Body pale brown, leaf-like, flattened, 18-51 mm. long by 4-13 mm. broad; the anterior 3-4 mm. forms a rather thick, conical portion which is followed by a large, flat, leaf-like body of elongate-oval form; this latter widens rapidly to the maximum breadth and then decreases gradually in width to the posterior end which is bluntly pointed; cuticle is covered with numerous spines placed side by side in alternating rows; oral sucker is anterior, round and terminal, but inclines ventrad; acetabulum about 3-4 mm. caudad of oral sucker, with which it closely agrees in size; genital pore median, about half way between oral sucker and acetabulum; œsophagus rarely over 1-1½ times as long as the pharynx; intestine dendritic; cirrus frequently extruded from pore and then recurved; testicles profusely branched, situated for the greater part posterior to transverse vitello-duct. Vulva at side of cirrus; uterus forms a rosette with its numerous coils, and is frequently visible to the naked eye as a dark brown spot immediately posterior to the acetabulum; ovary branched, anterior of transverse vitello-duct; vitellogene glands profusely branched, and occupy the entire margin of the body from acetabulum to posterior extremity; they lie dorsally as well as ventrally of the intestine, becoming wider posteriorly. Oviparous.

*Eggs* oval, 0.13-0.14 mm. long by 0.075-0.09 mm. broad; *miracidium* conical, ciliated, with oral papilla, two cup-shaped eye-spots, and rudimentary intestine; metamorphosis (sporocyst, redia, cercaria) takes place in small snails of the genus *Limnæa* (*L. truncatula* and others); *cercaria* whitish, owing to excessive development of the capsule glands; encysts upon plants.

### The Intermediate Hosts of F. hepatica.

PLATE IV.

It was shown by Leuckart and Thomas that in Europe the intermediate host for this fluke is a small swamp-snail, *Limnæa truncatula*; Leuckart also showed that the rediæ (but not the cercariæ) would develop in the young of another species of snail, *L. peregra*, and quite recently Lutz ('92 and '93) has shown that in Oahu and Kani (Sandwich Islands) two other snails may serve in this capacity, *i. e.*, *L. oahuensis* Souleyet and *L. rubella* Lea. In the case of *L. oahuensis*, Lutz states that "the infection can take place only in young specimens." None of these four very closely allied species are recorded for America, and yet we find *F. hepatica* in both North and South America, so that we must either have on this continent some other species of snail which may act as intermediate host, or some of the species described in America must be identical with some of the above-named forms.

The forms which would especially fall under suspicion are *L. humilis* Say in North America, and *L. viator* Orb. in South America.

This report is not the place to discuss the question as to whether *L. truncatula*, *L. peregra*, *L. oahuensis*, *L. rubella*, *L. humilis* and *L. viator* represent six well established species or not, as that is a matter for conchologists to decide; suffice it to say that specialists in conchology have described snails under these names; that the forms are all so very closely related, that a zoölogist would not commit a very grave offense against systematic zoölogy if he were to consider them as varieties of two or three species; that the forms described under the names *L. truncatula*, *L. oahuensis* and *L. rubella* are known to serve as intermediate host for the parasite now under discussion; that in Europe the rediæ (but not the cercariæ) develop in *L. peregra* and that it is probable, though not demonstrated as yet, that *L. humilis* is intermediate host for North America and *L. viator* for South America.

As these snails form the intermediate host of a dangerous, in

PLATE IV.

INTERMEDIATE HOSTS OF FASCIOLA HEPATICA.

Figs. 1 *a–c*. *Limnæa oahuensis*, after Souleyet. Fig. *c* nat. size, *a–b* enlarged.
Figs. 2 *a–c*. *L. viator*, after d'Orbigny. Nat. size and enlarged.
Figs. 3 *a–b*. *L. peregra*, after Lkt. Nat. size and enlarged.
Figs. 4 *a–b*. *L. truncatula*, after Lkt. Nat. size and enlarged.
Figs. 5 *a–j*. *L. humilis*, after Binney. Nat. size and enlarged.

many cases fatal parasite, they must be included among the worst enemies of the stock-raiser. On that account, and since our experiments with *F. magna* will be greatly influenced by the facts known in regard to the development of *F. hepatica*, the intermediate hosts in all probability being closely allied animals, it has been thought best to give the descriptions, etc., of all the snails which are known or supposed to act as intermediate hosts for *F. hepatica*.

The systematic position, and at the same time a certain amount of the anatomy of these mollusks may be seen from the following synopsis, based upon the writings of Gray ('57), Binney ('65), Souleyet ('52), Lea ('41), Jeffreys ('62), d'Orbigny ('35), Westerlund ('85) and others.

J. E. Gray.—Manual of the Land and Fresh-Water Shells of the British Islands. London, 1857.

W. G. Binney.—Land and Fresh Water Shells of North America. Part II. Smithsonian Misc. Collections, 143. Washington, 1865.

Souleyet.—Voyage autour du Mond, sur la Corvette La Bonite. Vol. II. 1852.

Lea.—On Fresh Water and Land Shells; Proc. American Phil. Soc. 1841.

Jeffreys.—British Conchology. Vol. I. 1862.

d'Orbigny.—Voyage dans L'Amerique Méridionale, 1835-1843.

C. A. Westerlund.—Fauna der in der Paläarctischen Region lebenden Binnenconchylien. II. 1885.

MOLLUSCA.—Class GASTROPODA. Mollusks with a distinct head, which is generally provided with tentacles and eyes; provided with a single median muscular foot with a broad sole, (*seldom with a laterally compressed fin or heel-like foot); the undivided mantle frequently secretes a spirally twisted (or plate-shaped shell); pallial cavity lateral and dorsal; mouth with jaws and tongue; respiration through lungs (or gills); hermaphroditic (or diœcious).

Order, PULMONATA.—Land or fresh water snails, (naked or) with shells; palial cavity lies on right side, as a rule anterior to heart, and is arranged for breathing air; true operculum absent; hermaphrodites.

Sub-order, BASOMMATOPHORA.—Eyes on the median side or at the base of the tentacles; tentacles can be contracted but not invaginated; labial tentacles absent; a well-developed external shell always present; genital openings separated.

" LIMNÆADÆ.—Animal with an elongate foot, a more or less conical spiral body, a short muzzle, with dilated lips and compressed tentacles, with the eyes near the inner side of their base;

*Statements in parentheses do not apply to *Limnæa*.

the mantle, which covers the body, has a thin edge and is protected by a variably shaped pale uniform colored shell, which is clothed with a hard olive periostracum" (Gray p. 196). Genital openings close to each other, the male opening nearer the tentacles, the female opening nearer the breathing pore; jaw composed of one or of several—3—pieces.

"They live in ponds and ditches, often floating on the surface of the water, their back downwards, or crawling on the mud at the bottom, or on aquatic plants, but always coming to the surface to respire." (Gray p. 196.)

"The family contains nine genera, which may be thus distinguished:

a. Shell ovate, spiral; pillar with an oblique plait.
  1. *Limnæus* [*Limnæa*]. Shell rough; inner lip simple.
  2. *Amphipeplea*. Shell polished, thin; inner lip expanded.

b. Shell conical, recurved; apex oblique.
  3. *Ancylus*. Apex of the shell to the right.
  4. *Velletia*. Apex of the shell to the left.

c. Shell ovate, sub-spiral; pillar smooth.
  5. *Otina*.

d. Shell ovate, spiral; pillar simple.
  6. *Physa*. Inner lip expanded.
  7. *Aplexus*. Inner lip not expanded.

e. Shell discoidal.
  8. *Planorbis*. Cavity of shell simple; mouth roundish or sub-quadrate.
  9. *Segmentina*. Cavity of shell divided by cross septa; mouth triangular.

"Tentacles short, compressed, triangular, without any auricle at the base; jaws 3, smooth; shell oblong, spiral. (*Limnæana*.)

1. LIMNÆA* (Mud Shell). "Animal with a short broad foot, broad short compressed tentacles, without any auricles at the base, a large upper and two small rudimentary lateral jaws, a large central spiral body, and a simple-edged mantle, covered by an external ovate, thin, dextral, transparent spiral-shell with an ovate mouth, having a single oblique plait on the middle of the column running into the axis.

"Limnæa has a small central tooth, as it were squeezed up between two very large lateral ones, each primary lateral having a very large apex internally, with a small external one, while at the edge they have altered to one thick prolonged apex projecting inwards and irregularly lobed on its upper edge." Gray, p. 199.

"The apex of the shell is often eroded or truncated; that is to

*The generic name has been spelt by authors in no less than nine different ways; but the correct orthography is undoubtedly *Limnæa* (from λιμναῖος—inhabiting marshes) as proposed by Rang.—Jeffreys, p. 101.

say, as the upper part of the body is withdrawn from the tip, and the body moves forwards into the larger part of the shell, it forms a septum behind, and the part that is thus separated eventually falls off." Gray, p. 200.

The Limnæi principally feed on the slimy matter which covers sticks, shells, and stones, beneath the water and on the mud, which is constantly found in the intestines. (Haldemann.)

Generic diagnoses of the genus Limnæa given by other authors differ slightly from the one just quoted; Gray's description will answer for all practical purposes, but as a comparison, and as the most recently revised diagnosis of the genus, the following is added from Westerlund ('85).

Gen. *Limnæa* (Brug.) Rang. Thier dick, Mundlappen vorn ausgerandet; die zwei Fühler zus.-gedrückt, dreieckig, kurz, mit den Augen innen an der Basis; Fuss keilfg., vorn abgestutzt, hinten spitz zugerundet; Mantel ganz eingeschlossen; Kiefer hornig, dreitheilig, mit breitem Mittelstücke u. schmalen, etwas gebogenen Seitenstücken; Zunge blattartig, vorn breit, hinten zus.-gelegt; die Mittelzähne s. klein, mit zwei Nebenhäckchen, die zahlreichen Seitenzähne grösser u. gesägt.

Gehäuse meist dünnschalig, mit Nabelspalt, ohrfg. o. eirund bis thurm o. spindelfg.; Umg. schnell zunehmend, der letzte oft s. weit u. fast das ganze Geh. bildend, dann das Gew. s. kurz, o. Gew. lang thurmfg. ausgezogen; Mund. weit, gerundet bis länglich eifg., Spindelsäule oft frei hervortretend, bogig, Spindelumschlag lamellenartig, anliegend, Mundsaum einfach, scharf.

Syn. 1753 *Auricula* Klein—1758 *Helix* pr. p. L.—1774 *Buccinum* pr. p. Müller—1778 *Turbo* pr. p. Da Costa—1791 *Lymnea* Brug.—1799. *Lymnæa* Lam. —1801 *Limneus* Drap.—1810 *Lymnus* Montf.—1815 *Lymneus* Biard —1817 *Limnæus* Cuv.—1826 *Limnea* Desh.—1829 *Limnæa* Rang --1841 *Lymnæus* Villa.

Subgenera:—

1. *Lymnus* Montf. Geh. festschalig, verlängert-eifg., mit gethürmtem, s. spitzem Gew., das meist von der Mündungslänge ist o. länger; Umg. typisch bei reifen Ex. 7-8, s. wenig gewölbt, anfangs langsam, dann s. rasch zunehmend, der letzte gross, aufgeblasen, m. o. w ausgezogen.—Sp. 1.

2. *Gulnaria* Leach. Geh. meistens dünnschalig, mit kurzem o. sehr kurzem, öfters zugespitztem Gew.; Umg. 4-5, der letzte s. gross u. aufgeblasen, den weitaus grössten Theil des Geh. einnehmend. Münd. s. gross, gerundet,—selten Geh. festschalig, eifg., mit kegelfgem Gew. u. spitzeifger oft weissgelippter Münd., Umg. 4-5, der letzte nur convex.—Sp. 2-6.

3. *Limnophysa* Fitz. Geh. meist dickschalig, verlängert eifg., mit konischen o. thurmfgem Gew., das meistens länger, zuweilen viel länger als die Münd. ist. Umg. 7-8, langsam zunehmend, der letzte ausgezogen, wenig gewölbt; Münd. meist mit zweifarbiger (nach innen weisser, nach aussen rothbrauner) Lippe.—Sp. 7.

4. *Leptolimnea* Swains. Geh. cylindrisch-thurmfg. Umg. 7-8, s. langsam zunehmend; Münd. klein, kaum ein Drittel der Gehäuselänge, innen meist mit glänzend weisser Lippe.—Sp. 8.

5. *Fossaria* W. Geh. klein, länglich-eifg., dünnschalig, mit m. o. w. offenem Nabelspalte; Gew. spitzkegelfg., etwas länger als die Münd.; Umg. 5-6, s. langsam zunehmend, stark gewölbt, der letzte meist auffallend weiter als die übrigen; Naht tief eingeschnürt.—Sp. 9-11.

6. *Tanousia* Bgt. Geh. klein, gedrungen, konischeifg., mit dem letzten Umg. erst s. aufgeblasen, dann allmählig verschmächtigt dass die Münd. s. eng wird; Münd. innen mit einer zus.-hängenden Lippe.—Sp. 12. Westerlund p. 23-24.

1. *Limnœa truncatula** (Müller). Pl. IV, Fig. 4. "Body dark brown or grey, of a lighter color on the lower side, covered with fine black specks, tentacles short, but slender, rounded at their tips; eyes nearly sessile: foot rather short, marked with milk-white spots, which are scattered and larger than the black specks, nearly truncate in front, gradually narrowing and abruptly rounded behind.

"Shell oblong-conic, turretted, rather solid for its size, glossy, yellowish-brown or horn colour; epidermis thin: whorls 5-6, rounded and convex, but compressed in the middle, so as to make the top of each appear somewhat truncate; the last whorl occupying about three-fifths of the shell: spire abruptly tapering to a rather fine point: suture extremely deep: mouth oval, scarcely contracted on the inner side: outer lip sharp: inner lip continuous with it and reflected on the columella, behind which is a distinct umbilical chink: fold rather slight but thick. L. 0.4. B. 0.2. (in)" (3.5-1.5 mm. long by 1.8-5 mm. broad).

"Var. 1. *major*. Shell larger: whorls more swollen and the last considerably exceeding the usual proportion of size.

"Var. 2. *elegans*. Shell much larger, more solid and slender, greyish-white, marked with coarse spiral ridges: spire much produced: suture oblique: outer lip thickened. L. 0.6. B. 0.225" (in). (15 mm. long by 5 mm. broad).

"Var. 3. *minor*. Shell much smaller, thinner and semi-transparent, dark horn colour, marked with stronger and closer longitudinal striæ. L. 0.285. B. 0.165." (in) (7 mm. long by 4 mm. broad).

"Var. 4. *albida*. Shell smaller and white."

"Var. 5. *scalariformis*. Shell smaller: whorls nearly disunited."

"Var. 6. *microstoma*. Shell smaller and narrower: whorls more swollen: mouth contracted." J. G. Jeffreys. '87. I. pp. 115-117.

This snail is found on the banks of slow and muddy rivers and streams, marshes, ditches, etc. It is nearly amphibious in its

---

* Synonymy:—*Buccinum truncatulum* Müller; *Helix fossaria; Lymnœus minutus* Drap.; *Lymnœus fossarius; Limmea fossaria; Limneus minutus; Limnœa truncatula.*

Westerlund gives the following specific description recognizing 25 varieties:—

*L.* (*Fossaria*) *truncatula.* Geh. dünn, feingestreift, horn-braun; Umg. m. o. w. wendeltreppenartig abgesetzt; Münd. eifg., oben stumpfeckig, in der Regel kürzer als das Gew., Mündungswand s. quer, Spindel fast gerade herabsteigend, Munds. gerade. G. 9: 4., M. 4 mm. (Europa, Nordafrica, Nord. u. Westasien).

habits, being found more frequently out of the water than in it. It deposits its spawn on the mud, which it generally inhabits, and not like its congeners on stalks and under the leaves of water-plants. It is found from Siberia to Algeria and Sicily, occasionally in elevated spots.

2. *L. peregra** (Müller). Puddle Mud Shell (Pl. IV, Fig. 3). "Body yellowish-grey, with a brown or olive green tinge, mottled with black and covered with small yellow or milk-white, and black specks: tentacles diverging from each other at nearly a right angle: eyes distinct: foot oblong, very broad, nearly truncate in front, and obtusely rounded behind."

"Shell obliquely ovate, thin, moderately glossy, semi-transparent, yellowish-horn colour, irregularly striate by the lines of growth, and closely and microscopically striate in a spiral direction, with occasionally a few indistinct spiral ridges and pitmarks: epidermis rather thin: whorls 5, convex, the last occupying three-fourths of the shell: spire produced and pointed: suture rather deep: mouth large, oval, very little contracted above by the projection of the penultimate whorl: outer lip thin, slightly reflected: inner lip folded on the columella and thickened, forming behind it a slight umbilical cleft: fold rather prominent and curved. L. 0.75. B. 0.425. (in.)" (8-20 mm. long by 4-10 mm. broad).

"Var. 1. *Burnetti.* Body a little broader than that of the typical form, dark olive, spotted with opaque yellow: mantle nearly black, with a few paler spots. Shell rather globular and solid, of a dull aspect, yellowish brown, closely and strongly striate in the line of growth: epidermis rather thick: the last whorl nearly covering all the others: spire exceedingly short, nearly truncate and almost intorted. L. 0.725 in. B. 0.65 in." (18 mm. long by 16 mm. broad.) Syn. *Limnæa Burnetti* Alder; *Limnæus Burnetti* F. & H.

"Var. 2. *lacustris.* Body of a darker colour than usual. Shell resembling that of the last variety, but it is much smaller and more glossy, and has strong and regular transverse grooves, and the spire is not quite so short nor inclined to be intorted. The shell is often eroded. Syn. *Gulnaria lacustris,* Leach."

---

**L.* (*Gulnaria*) *peregra* Westerlund.

Synonymy after Gray ('57): *Buccinum peregrum* Müller; *Bulimus pereger* Brug.; *Helix peregra* Gm.; *H. putris* Penn.; *Lymnæa putris* Flem.; *Limnæus pereger* Drap.; *Lymnæa peregra* Lamarck; *Gulnaria peregra* Leach; *Lymnæus vulgaris* Pfeiffer; *Limnæus opacus* Ziegler; *L. fuliginosus* Z.; *L. callosus* Z.; *L. consobrinus* Z.; *L. nitidus* Z.; *L. corneus* Z.; *L. solennis* Z.; *Buccinum rivale* Studer; *Limnæa limosa* and *peregra* Mog.-Tand.; *Limnæus fontinalis* Stud.; *Limnea intermedia* Ferus; *L. thermalis* Boub.; *L. Nouletiana*, and *Frencaleonis* Gass.; *L. glacialis* Dupuy; *Turbo trianfractus* Da Costa; *Helix inflata* Gm.; *H. teres* Gm.; *H. siculus* Dillw.; *H. auricularia* B. M. & R.; *Bulimus siculus* Brug.; *H. auricula junior* Dillw.; *Lymnæa intermedia* Lam.

"Var. 3. *lutea.* Shell remarkably solid, having a very short spire of 3-4 whorls. Syn. *Helix lutea.*"

"Var. 4. *ovata.* Body of a paler colour. Shell ampullaceous and rather thinner than usual: whorls exceedingly convex, the last being larger in proportion to the rest: spire very short: suture deep: mouth very large. Syn. *Limneus ovatus* Drap."

"Var. 5. *acuminata.* Shell resembling the last variety in all respects, except in having a more produced spire and a smaller mouth."

"Var. 6. *intermedia.* Shell rather compressed towards the front margin and thinner than usual: spire more produced: mouth expanded. Syn. *Limnea intermedia* Fer."

"Var. 7. *oblonga.* Shell oblong and compressed in front."

"Var. 8. *labiosa.* Shell smaller, having the outer lip remarkably expanded and reflected. L. 0.5. B. 0.35 in." (12.5 mm. long by 8.75 mm. broad.)

"Var. 9. *picta.* Shell rather smaller than the last, and beautifully marked by alternate bands of brown and white, which are sometimes confluent."

"Var. 10. *maritima.* Shell dwarfed, rather solid: spire produced: suture deep. L. 0.4. B. 0.225 in." (10 mm. long by 5.6 mm. broad.)

"Var. 11. *Succineæformis.* Shell shaped like a Succinea, and very thin: whorls 4: spire small and oblique."

"Var. 12. *decollata.* Shell more or less eroded: spire truncate."

"Var. 13. *sinistrorsa.* Shell resembling a *Physa* in having the spire sinistral or reversed, rather solid: the spiral ridges distinct and prominent. Syn. *Limnæus lineatus.*"

"Var. 14. *scalariformis.* Shell oblong, with deep and regular transverse striæ; whorls more or less disjoined: suture consequently very deep." Jeffreys p. 104-108.

This species is very widely distributed, being found from Siberia to Sicily. It lives in still or slowly running waters. It is nearly amphibious and may be met with some distance from the water. It is very prolific, laying about 1300 eggs in a season, the eggs being in clusters of 12-180. *L. peregra* is both zoö- and phytophagous, and is extremely variable, and no less than 30 species have been made out of its varieties.

3. *L. humilis** Say (Pl. IV, Fig. 5).—"Shell ovate-conic, thin, translucent, with slight wrinkles; volutions nearly six, convex, terminal one very minute; suture well indented; aperture about equal in length to the spire; labium with an obvious plate of calcareous deposit; a distinct and rather open umbilical aperture; color pale reddish-white or yellowish-white. Total length seven-twentieths of an inch (8.75 mm.). Ranges from Maine to Georgia and Kansas to Lake Superior." W. G. Binney. Land and Fresh Water Shells of North America, 1865, p. 65.

*Synonymy after Binney: 1822, *L. humilis* Say; 1825, *L. modicella* Say; 1841, *L. parva* Lea; 1841, *L. plica* Lea; 1841, *L. griffithiana* Lea; 1841, *L. planulata* Lea; 1841, *L. rustica* Lea; 1841, *L. exigua* Lea; 1841, *L. curta* Lea; 1843, *L. linsleyi* de Kay.

4. *L. oahouensis** Souleyet. (Pl. IV, Fig. 1.)—Shell oblong-conic, wound right or left, thin, nearly translucid, of a tawny brown color, sometimes covered with a black and persistent coating; spire conical, generally eroded at the summit; whorls 4-5, depressed convex; mouth oval; internal lip reflected and adhering to the second last whorl; edge thin and sharp. Foot short and rather abruptly pointed posteriorly; the anterior border of the head slightly indented in the median line; tentacles are short, flattened, straight and terminated in a sharp point. Body is black.

Shell 12 mm. long by 9 mm. broad.

5. *L. rubella*† Lea. Body darker than that of *L. oahuensis;* feelers longer and more filiform, but thicker at the base, where they are triangular; teeth of radula similar to those of *L. oahuensis.* Shell sinistral, ovate-conic, thin, light and translucent with a reddish tinge; spire is short, suture shallow; whorls 5, convex. Aperture oval, 7.5 mm. Length 13 mm., breadth 6.5 mm.

The determination of the snails from the Sandwich Islands seems to be very difficult and the persons who have determined

---

*The original description reads as follows:

Lymnaea. testâ oblongo-conicâ, dextrâ vel sinistrorsâ, tenui, subpellucidâ, fuscâ, interdùm nigratâ; spirâ conicâ; anfractibus 4-5; convexo-depressis; apice saepiùs eroso; aperturâ ovatâ; labio reflexo, adnato; labro tenui, acuto.

Coquille oblongue conique, dextre ou sénestre, mince, subtranslucide, d'un brun-fauve, parfois couverte d'un enduit noirâtre et persistant. Spire conique, à sommet le plus souvent rongé, et composée de quatre à cinq tours d'une forme convexe-deprimée. Ouverture ovale; lèvre interne réfléchie et adhérente à l'avant-dernier tour; labre mince et tranchant.

Cette espèce présente, comme nous venons de le dire, la particularité d'être tantôt dextre et tantôt sénestre. L'animal a le pied court et assez brusquement rétréci en pointe à sa partie postérieure; le bord antérieur de la tete est légèrement échancré sur la ligne médiane; les tentacules sont courts, aplatis, étroits et terminés en pointe aigue. Toutes ces parties sont d'un gris moirâtre.

Dimensions de la coquille.—Longueur, douze millimètres; largeur, au dernier tour, neuf millimètres.

Cette Lymnée habite les ruisseaux de l' île Oahu (îles Sandwich); elle y est très commune." (Souleyet, Voyage autour du Monde, sur la Corvette La Bonite, Vol. II, p. 527. Pl. 29. Figs. 38-41. 1852.)

†Original diagnosis:—

*Lymnea rubella.*—Testâ ovato conicâ, tenui lævi, nitidâ, diaphanâ, rubella, imperforata; spirâ breviusculâ; suturis parvis; anfractibus quinis, subconvexis; aperturâ subgrandi, ovatâ. *Hab.* Oahu. (Lea. On Fresh Water and Land Shells; Proc. Amer. Phil. Soc. 1841, p. 31.)

the specimens which Lutz describes have arrived at different results, as shown by the following table :

| Lutz. | Böttcher. | Baldwin. | Streng. |
|---|---|---|---|
| *L. pereger*<br>L. No. 2. | *L. oahuensis* Soul. | *L. turgitula* Pease. | *L. umbilicalis* Mögh. |
| L. No. 4. | | *L. oahuensis* Soul. | *L. rubella* Lea. |
| L. No. 5. | | *L. rubella.* | *L. sandwichensis* Phil. |

Lutz's species Nos. 2 and 4 are the forms which serve as hosts for *F. hepatica.* To establish the proper synonymy of these animals does not come within the province of this report.

6. *L. viator** d'Orb., 1835 Body viridescent. Shell oblong, elongate, somewhat ventricose, very slightly umbilicate. thin, fragile, smooth or marked with very light lines of growth; spire more or less elongated, conical, very sharp at the tip; whorls 5, very detached, convex, separated by deep suture; mouth oval or almost round, with thin lips; columella curved, occasionally rather sinuous. Color, a uniform tawny gray. 8 mm. long by 4 mm. broad.

I am indebted to Dr. Dall of the Smithsonian Institution for the use of his private library in compiling the above data on these snails.

*D'Orbigny's description reads as follows:

L. corpore viridencente.

Testâ elongato-oblongâ, subventricosâ, subumbilicatâ, laevigatâ, tenui, livido-fuscescente; spirâ subelongatâ, conicâ, apice acuto, anfractibus quinis convexis; suturâ profundâ; aperturâ ovali; labro acuto. Long 8 mm.; lat. 4 mm.

Coquille. Oblongue, allongée, un peu ventrue, très légerèment ombiliquée, mince, fragile, lisse ou marquée de très légères lignes d'accroissement; spire plus ou moins allongée, conique, à sommet très-aigu; composée de cinque tours très détachés, fortement convexes, séparés par une suture très profonde; bouche ovale ou presqu-arrondie, à bords minces; columelle arquée, quelque fois un peu sinueuse. Couleur: gris-fauve uniforme.

Cette coquille varie un peu selon les localités: aux environs du Callau, au Pérou, elle est plus allongée, à tour plus détaillés; tandis qu' en Patagonie et au Chili, elle est un peu ventrue, et ses tours sont moins convexes. Au premier aperçu, nous l'avons concidérée comme une simple variété du *Limnæus minutus*, de France; mais en les comparant avec le plus grand soin, nous avons reconnu que notre *L. viator* est toujours moins allongé à proportion, beaucoup moins ombiliqué, et qu'il était de plus presqu' entièrement lisse; tandisque le petit Limnée es un peu strié; néanmoins, il est peu d'espèces qui aient plus de rapports entr'-elles.

Nous avons rencontré cette espèce en Patagonie, au 41 degré de latitude sud, sur les rives de Rio negro, à commencer de sept ou huit lieues au-dessus de son embouchure, jusque bien avant sur son cours; il y est très commun. Nous l'avons retrouvé ensuite au Chili, aux environs de Santiago et de Casa blanca, toujours dans les ruisseaux d'eau limpide. Lorsque, plus tard, nous avons recherché les mollusque des environs de Lima, nous avons encore recueilli cette espèce dans tous les canaux d'irrigation qui sortant du Rimac entourent la ville de Lima et celle du Callao; mais dans ces deux localités, tous les individus sout constamment plus allongés, a tours plus séparés que celle du Chili, et de Patagonie; et, vu la différence de lieu d'habitation, nous aurions été tenté d'en former deux espèces distinctes, si nous n'avions craint de trop multiplier les espèces ; et si, d'ailleurs nous n'eussions pas reconnu que les individus des environs de Lima étaient aussi moins allongés, que ceux du Callao. Dès-lors, nous avons dû croire que des circonstances locales seules avaient influé sur ce léger changement de Formes. (d' Orbigny, Voyage dans l'Amerique Meridionale, p. 340. Plate 43. Fig. 1-3 ).

(*To be Continued.*)

## Figures and Tables

**Plate III. f1:**
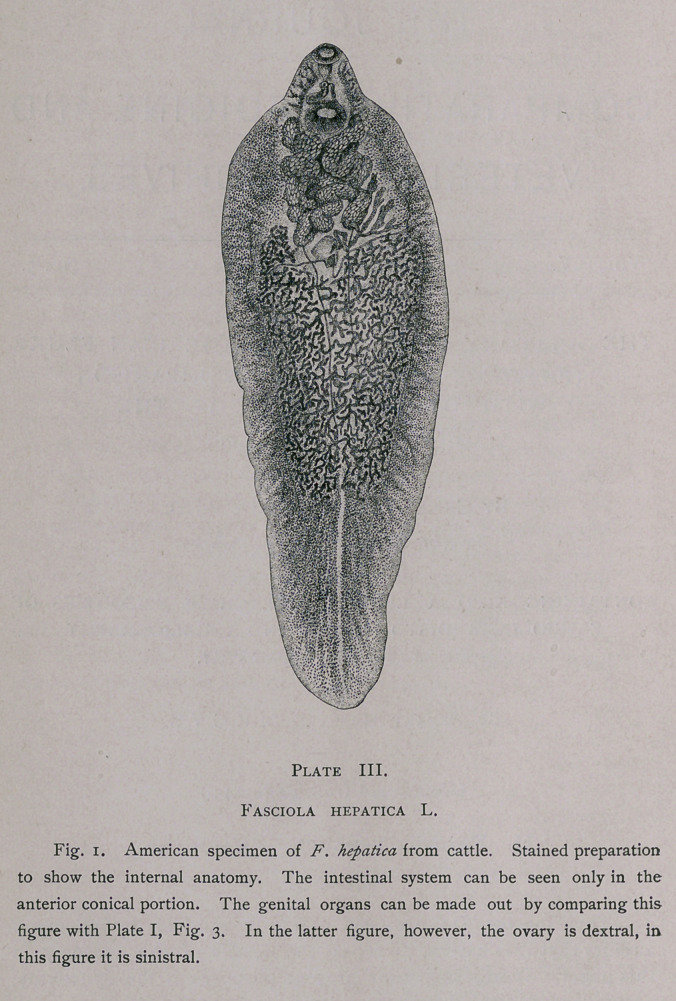


**Plate IV. f2:**